# Genotypic and phenotypic characterization of rare globin variants in Northern Guangxi, China

**DOI:** 10.3389/fimmu.2025.1695120

**Published:** 2025-10-28

**Authors:** Wei-Jia Yang, Qing-Ping Kang, Li-Ming Liang, Qian Zhou, Xiao-Min Gong, Min Dou, Cui-Juan Huang, Ying Lin

**Affiliations:** 1 Department of Eugenics and Genetics, Guilin People’s Hospital, Guilin, Guangxi, China; 2 Genetic Metabolism Laboratory, Guilin Women and Children’s Hospital, Guilin, Guangxi, China; 3 Genetic and Precision Medicine Lab, The First Affiliated Hospital of Guilin Medical University, Guilin, Guangxi, China

**Keywords:** hemoglobin electrophoresis, phenotype, rare globin variation, genotype thalassemia, molecular diagnosis

## Abstract

**Objective:**

The aim of this study is to examine the relationship between hematological parameters, hemoglobin electrophoresis findings, and phenotypic characteristics in individuals carrying rare thalassemia gene variants in Northern Guangxi, China.

**Methods:**

Peripheral blood samples were collected from 3,890 individuals (including 834 couples) who tested positive for thalassemia at the Prenatal Diagnosis Center of Guilin People’s Hospital between March 2019 and March 2025. Standard thalassemia genotyping was performed using Gap-PCR and PCR-reverse dot blot (PCR-RDB) assays to detect common α- and β-thalassemia mutations prevalent in southern China. Participants with negative results genotype-phenotype discordance underwent extended molecular testing to detect rare thalassemia variants. In cases where both partners were identified as carriers, amniotic fluid samples were collected from pregnant women for prenatal diagnosis.

**Results:**

Thalassemia major was diagnosed in 13 fetuses, with elective termination of two affected pregnancies. The detection rate for common thalassemia mutations was 44.27% (1,722/3,890), while rare variants were identified in 1.72% (67/3,890). Among participants with negative results from conventional genotyping, the detection rate of rare mutations increased to 26.38% (67/254). A total of 42 rare thalassemia variants were found, including 25 α-thalassemia, 14 β-thalassemia, and 3 δ-thalassemia mutations. A novel 4.3 kb deletion (chr16:176935–181274DEL), encompassing the *α*1 gene and a recombined non-functional gene-X-Y-Z segment, was reported for the first time. The *-*α^4.3^/–SEA genotype was associated with HbH disease.

**Conclusion:**

A substantial frequency of rare thalassemia gene mutations was identified in the Northern Guangxi population, contributing to the regional mutational landscape. These rare genotypes were associated with distinctive hematological and hemoglobin electrophoretic features. Characteristic phenotypic patterns, combined with specific laboratory parameters, facilitated preliminary inference of genotypes and supported the application of targeted diagnostic approaches. This strategy may improve diagnostic accuracy, reduce missed or incorrect diagnoses, and enhance prenatal and postnatal management strategies.

## 1 Introduction

Thalassemia is the most prevalent and clinically significant monogenic disorder worldwide. It is characterized by hemolytic anemia resulting from deletions or mutations in genes involved in globin chain synthesis, which disrupt the balance of α- and β-globin production and subsequently shorten erythrocyte lifespan. In China, approximately 30 million individuals are carriers of thalassemia-related genetic variants, with an estimated 300,000 individuals affected by thalassemia major (TM) or thalassemia intermedia (TI), both of which require clinical intervention (1). Carriers generally exhibit normal life expectancy and development; however, offspring of two carriers have a 25% risk of inheriting TM or TI.

Management of TM typically necessitates long-term blood transfusion and iron chelation therapy, contributing to significant financial, temporal, and physical burdens. Although hematopoietic stem cell transplantation represents a potentially curative option, its application is limited by high cost and restricted availability. Consequently, preventive strategies remain central to disease control. Since 2010, the government-initiated “Guangxi Thalassemia Prevention and Control Plan” in Guangxi, China, has adopted an integrated approach comprising free premarital screening, pre-pregnancy assessment, prenatal screening, and prenatal diagnosis. Accurate thalassemia genotyping serves as a cornerstone of this program.

Conventional genetic testing methods for thalassemia, such as reverse dot blot hybridization, have been routinely used in clinical practice for over two decades. These methods facilitate the detection of 3 common α-thalassemia deletions, 3 *α-globin* point mutations, and 18 *β-globin* point mutations. However, a proportion of thalassemia cases remain undiagnosed using these standard approaches. Currently, over 130 α-thalassemia and more than 300 β-thalassemia mutation types have been identified (2). Tang et al. examined 72 participants suspected of carrying rare thalassemia mutations and identified uncommon *α*- or *β*-*globin* gene variants in 49 cases through a combination of next-generation sequencing (NGS), third-generation sequencing (TGS), and chromosome microarray analysis/copy number variation (CNV) sequencing (3).

Yin et al. reported 5 rare thalassemia cases among 20 samples using TGS (4). Zhuang et al. conducted genetic screening in a cohort of 6,174 participants, identifying 2,390 carriers (38.71%) of *α*- or *β*-*globin* gene mutations, including 40 individuals with rare or novel variants (5). Peng et al. developed a TGS-based method known as Comprehensive Analysis of Thalassemia Alleles, which successfully identified 10 clinically relevant variants including 3 structural variants and 7 single nucleotide variants among 100 participants who presented with abnormal hematologic parameters or hemoglobin electrophoresis results but had negative findings on standard genetic tests (6). These studies used methods such as GAP-polymerase chain reaction (PCR), NGS, TGS, and other molecular techniques to improve the detection of rare thalassemia mutations.

Guilin, located in northeastern Guangxi, is recognized as a region with a high prevalence of thalassemia. Guilin People’s Hospital has been conducting thalassemia genetic and prenatal diagnostic testing for the past six years. The subsequent section outlines the findings of the institution from its screening for rare thalassemia variants.

## 2 Participants and methods

### 2.1 Study participants

Group A included 3,890 participants who provided peripheral blood samples at the Prenatal Diagnosis Center of Guilin People’s Hospital between March 2019 and March 2025. Participants ranged in age from 2 to 48 years and comprised of 1,826 males and 2,064 females. Among these, 834 samples were from couples undergoing joint screening.

Group B included 13 participants (9 males and 4 females) aged 3 to 44 years, who were identified through retrospective data review conducted during the same period. These participants presented with clinical features indicative of thalassemia but exhibited discordant findings on conventional genotyping.

Group C consisted of 834 couples who underwent routine thalassemia genotyping at the same center within the same timeframe. For couples in which both partners were identified as thalassemia carriers, prenatal diagnostic testing was recommended. In such cases, amniotic fluid samples were collected from the pregnant women for fetal thalassemia gene analysis.

#### 2.1.1 Inclusion criteria

(1) Positive thalassemia screening with negative findings on conventional genotyping was defined by the presence of at least one of the following six criteria: 1) mean corpuscular volume (MCV) < 82 fL; 2) mean corpuscular hemoglobin (MCH) < 27 pg; 3) HbA2 < 2.4% on hemoglobin (Hb) electrophoresis; 4) HbA2 ≥ 3.5%; 5) elevated fetal hemoglobin (HbF) levels (typically ≥ 2%); or 6) abnormal hemoglobin profiles. Cases meeting any of these parameters were classified as screen-positive.

(2) Cases with a prior clinical diagnosis of TM or TI, presenting with mild anemia but negative results on conventional genotyping, were re-evaluated. Participants who continued to test negative upon retesting were subsequently included for further analysis of rare thalassemia gene variants.

#### 2.1.2 Exclusion criteria

Cases involving iron deficiency anemia, immune-mediated hemolysis, or other hematologic disorders were excluded from the analysis.

### 2.2 Informed consent selection

The study protocol was approved by the Ethics Committee of Guilin People’s Hospital (approval numbers 2022-072KY, 2023-120KY). Written informed consent was obtained from all participants. For participants under 18 years of age, consent was provided by a legal guardian.

### 2.3 Laboratory methods

#### 2.3.1 Analysis of blood routine parameters

Whole blood cell analysis was conducted using the Sysmex XE-5000 fully automated hematology analyzer.

#### 2.3.2 Hb electrophoresis detection and analysis

HB component quantification was conducted using the Sebia Capillarys 2 Flex Piercing system, a fully automated capillary electrophoresis platform.

#### 2.3.3 Serum ferritin detection

SF levels were measured using the Roche cobas e801 automated chemiluminescence immunoassay system.

#### 2.3.4 Thalassemia gene detection

##### 2.3.4.1 Conventional thalassemia gene detection

Genomic DNA was extracted from peripheral blood and amniotic fluid samples using a nucleic acid extraction kit provided by Shenzhen Yilifang Biotechnology Co., Ltd. A total of 25 common thalassemia genotypes were assessed. GAP-PCR was used to find four α-thalassemia deletions: αα/-α*^3.7^*, αα/-α*^4.2^*, αα/–*^SEA^*, and αα/–*^Thai^*. RDB-PCR was applied for the identification of three *α-globin* point mutations: αα/αα*^CS^*, αα/αα*^WS^*, and αα/αα*^QS^*.

Eighteen β-thalassemia mutations were also analyzed, including CD41–42, CD43, IVS-II-654, IVS-II-28, IVS-II-29, IVS-II-30, IVS-II-32, CD71–72, βE, CD17, CD31, CD37, CD14–15, CD27–28, IVS-I-1, IVS-I-5, CAP + 1, and IntM.

Samples suspected of carrying rare thalassemia variants were referred to third-party laboratories for further analysis, including Yaneng Bioscience Co., Ltd., Shenzhen Yilifang Biotechnology Co., Ltd., BGI Genomics, and Beijing Berry Genomics Biotechnology Co., Ltd.

##### 2.3.4.2 Rare thalassemia detection technologies

The following methods were used for the detection of thalassemia gene variants:

(1) Nested PCR;
(2) Multiplex ligation-dependent probe amplification (MLPA);
(3) Sanger sequencing (first-generation sequencing);
(4) NGS;
(5) TGS;
(6) Long-read index PCR based on the novel CycloneSEQ nanopore sequencing platform (Shenzhen BGI Genomics). Long-read sequencing was conducted using the CycloneSEQ WT-02 single-molecule nanopore sequencer, developed by BGI Genomics. This protocol, approved by the BGI Genomics Institutional Review Board (IRB24094), enables comprehensive detection of genetic variants in the HBA, HBB, HBD, and HBG loci, including single nucleotide variations (SNVs), deletions, and structural variations.

## 3 Results

### 3.1 Results from Group C – prenatal diagnosis in carrier couples

Among the 834 couples screened, 93 were identified as carrying genetically identical thalassemia genotypes. Prenatal diagnosis was performed in 48 couples, each involving a pregnant woman carrying a fetus at risk, while data from the remaining carrier couples who underwent testing at external institutions were excluded from analysis. No rare genotypes were identified among the 48 couples. TM was diagnosed in 13 fetuses, with the corresponding genotypes and pregnancy outcomes presented in **Table 1**.

**Table 1:** Prenatal genotype diagnosis of 13 fetuses with thalassemia major.

Category	Genotype	N	Pregnancy outcome
α-thalassemia	–SEA/–SEA	1	Termination of pregnancy
	-α3.7/–SEA	5	Termination of pregnancy (n=3), continued pregnancy (n=2)
	-α4.2/–SEA	1	Termination of pregnancy
	ααWS/–SEA	4	Continued pregnancy
β-thalassemia	βCD41-42/βCD41-42	1	Termination of pregnancy
α-/β- thalassemia	αα/ααCS with βCD41-42/β-50	1	Continued pregnancy

### 3.2 Results from Group A – routine genotyping and identification of discordant cases

Among the 3,890 participants in Group A, routine thalassemia genotyping of peripheral blood samples identified 1,081 cases of α-thalassemia, 551 cases of β-thalassemia, and 90 cases with combined α- and β-thalassemia mutations, resulting in an overall detection rate of 44.27% (1,722/3,890). The remaining 2,168 participants tested negative for common thalassemia genotypes. Following the exclusion of 1,375 cases associated with hematological conditions such as infections and iron deficiency anemia, 763 cases remained with negative results on routine genotyping but positive outcomes on thalassemia screening, representing 19.61% (763/3,890).

### 3.3 Rare thalassemia genotyping in discordant and referred cases

Of the 763 cases with negative results on routine genotyping but positive thalassemia screening, all individuals were contacted by telephone and invited to participate in rare thalassemia gene testing. A total of 241 participants provided informed consent. Including 13 additional cases from Group B, a total of 254 participants underwent rare thalassemia genotyping, resulting in the identification of 67 cases (refer to **Supplementary Tables 1-7**). The detection rate for rare thalassemia mutations in this cohort from northern Guangxi was 1.72% (67/3,890), with a detection rate of 26.38% (67/254) among those who tested negative for common genotypes. A total of 42 rare genotypes were identified, consisting of 25 α-thalassemia, 14 β-thalassemia, and 3 δ-thalassemia variants.

#### 3.3.1 Copy number variations

**Figure 1:**
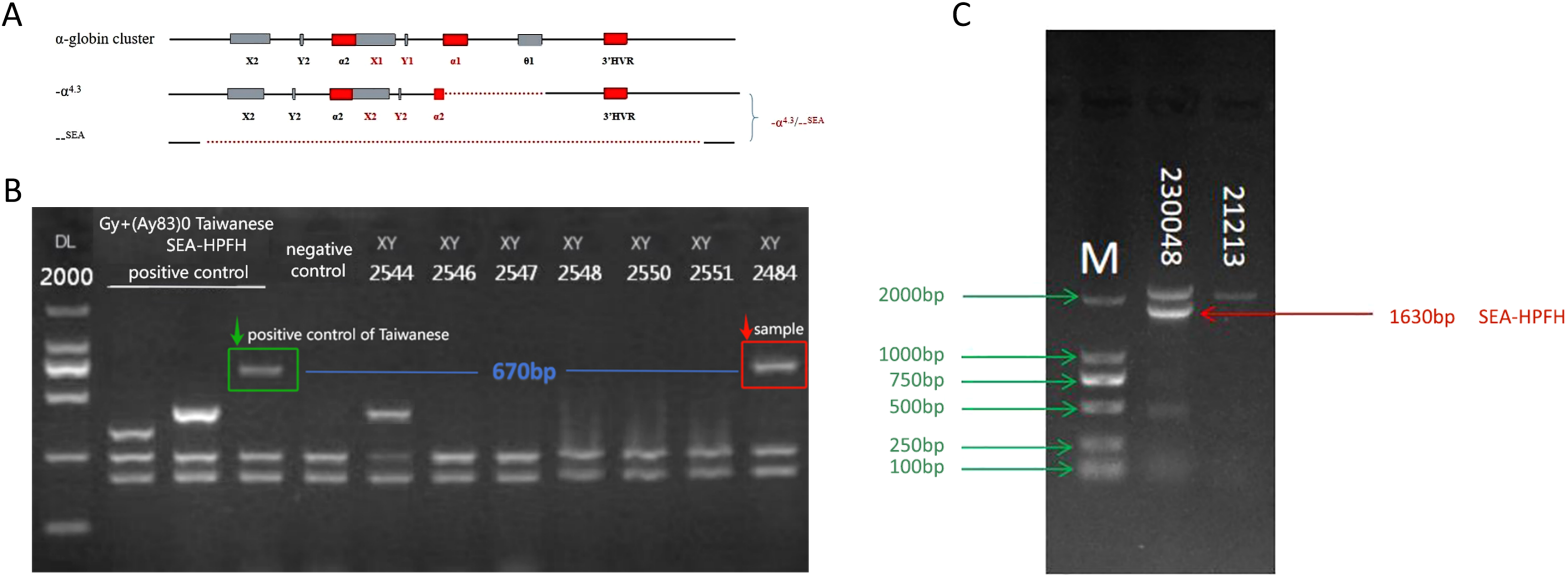
Rare CNVs in the *α-* and *β-globin* gene clusters. **(A)** −α^4^.^3^/−−SEA deletion (rare 4.3& kb deletion: chr16:176935–181274DEL) involving the *α1* gene and a non-functional X–Y–Z segment. **(B)** Sample 2484: GAP-PCR agarose electrophoresis showing a 670& bp band consistent with the positive control for the Taiwan-type deletion. **(C)** Sample 230048: GAP-PCR agarose electrophoresis revealing a 1630& bp band indicative of SEA-HPFH deletion.

Five rare CNV types involving the *α*- and *β*-*globin* gene clusters were identified, encompassing eight cases (excluding Thailand-type deletions due to coverage by the detection kit). Among these, the *α*-*globin* cluster included the α2.4 deletion, the 4.3 deletion (**Figure 1A**), the family-α90_93(-8bp)(AGCTTCGG) variant, and the family-HS-40 deletion, as well as one duplication event. Within the *β-globin* gene cluster, two rare CNVs were found: one of Taiwan-type heterozygosity (**Figure 1B**) and one case of Southeast Asian (SEA) deletion with heterozygosity involving the SEA type (**Figure 1C**).

#### 3.3.2 Rare SNVs in the *α-globin* gene cluster

**Figure 2:**
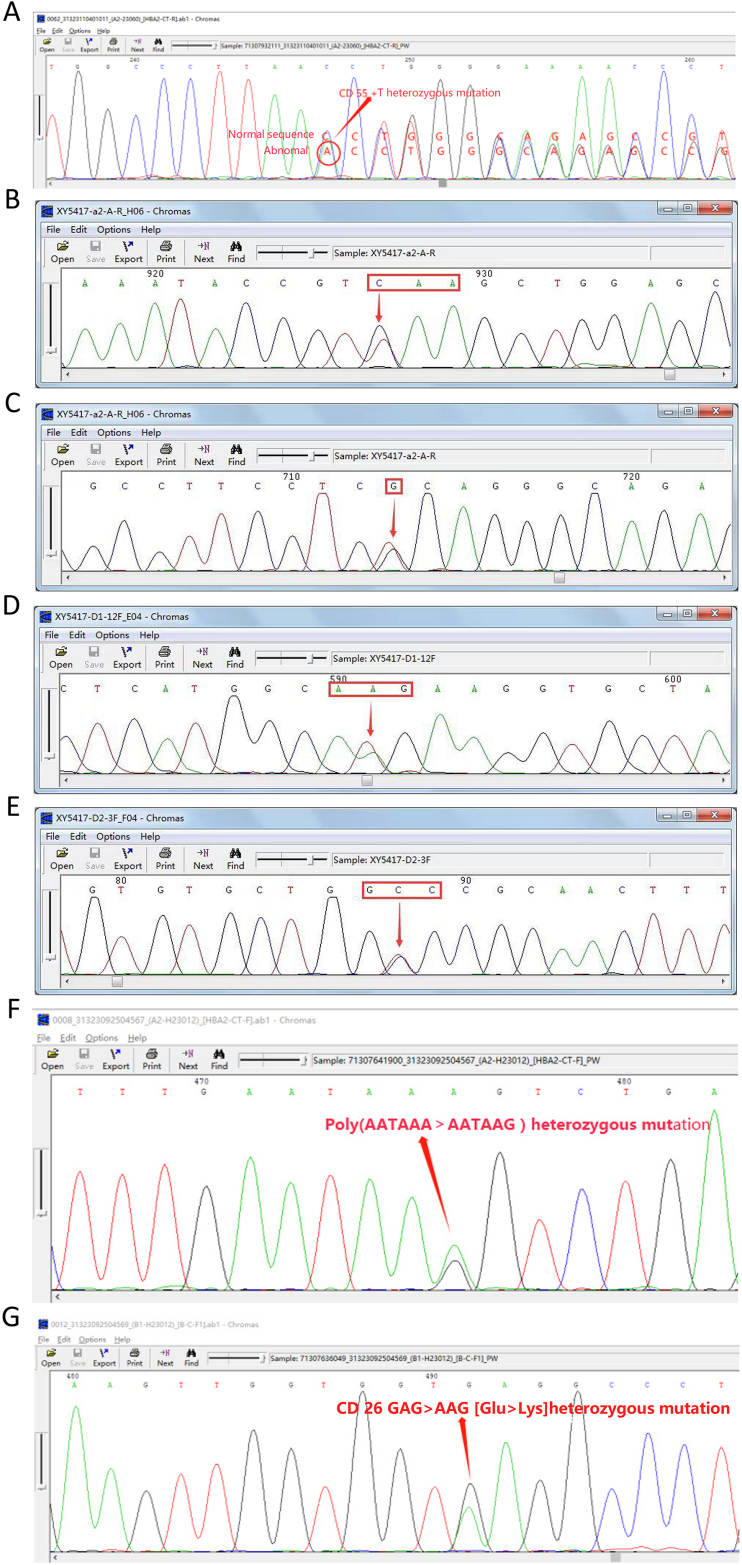
Seven rare SNVs in the *α-globin* gene cluster. **(A)** Codon 55 insertion: *HBA2:c.168dup* (αα/ααHBA2:c.168dup). **(B)** Nonsense mutation: CS (TAA > CAA). **(C)** IVS-II-55 (T > G). **(D)** CD65 (AAG > ATG). **(E)** CD115 (GCC > GTC). **(F)** PolyA signal mutation: *HBA2:c.*94A > G* (AATAAA > AATAAG). **(G)** β-globin mutation: *βCD26* sequencing diagram.

Seven rare SNVs were identified within the *α-globin* gene cluster. These included ααCD30 (*HBA2*: c.91_93delGAG) with SEA heterozygosity, *HBA2*: c.168dup (**Figure 2A**), and three intronic variants: IVS-I-117 (G > A), IVS-II-34 (G > A), and IVS-II-55 (T > G). Among these, IVS-II-55 (T > G) was observed in one case coexisting with both the α-thalassemia Constant Spring (CS) mutation and δ-thalassemia (**Figures 2B–E**). Additionally, one case involved a PolyA signal mutation (AATAAA > AATAAG) co-occurring with β-thalassemia (**Figures 2F–G**).

#### 3.3.3 Rare SNVs in the *β-globin* gene cluster

Five rare SNVs were detected within the *β-globin* gene cluster. These included three cases of β^n^/βCD30 (A > G), one case of β^n^/β−31 (A > C), and three intronic variants: IVS-II-5 (G > C), IVS-II-81C, and IVS-II-672 (A > C).

#### 3.3.4 Hong Kong-type and triplicated *α-globin* genotypes

Hong Kong-type and triplicated *α-globin* genotypes were identified and included three cases of HKαα/αα, one case of HKαα/αα*^CS^*, three cases of HKαα/αα*^WS^*, and one case of HKαα/−−*^SEA^*. Two triplicated α-globin arrangements were detected: two cases of β*^41–42^*/ααα*^aaa3.7^* and two cases of β*^17^*/ααα*^aaa4.2^*.

#### 3.3.5 Structural hemoglobin variants in the *α-globin* cluster

**Figure 3:**
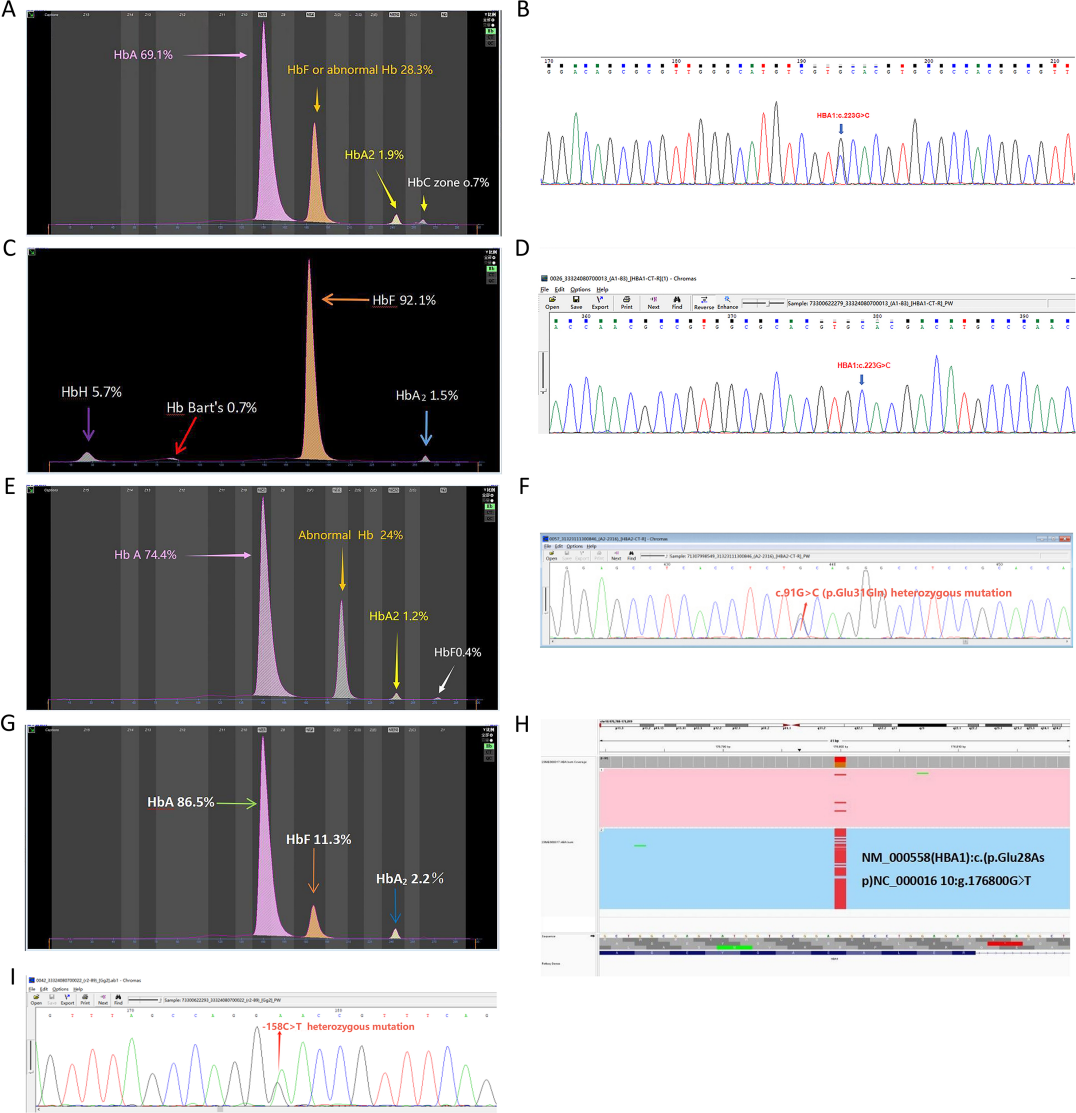
Rare hemoglobin structural variant genotypes in the *α-globin* gene cluster. **(A)** Electrophoresis pattern of *αα/−α^4^.^2^Hb Q-Thailand* (*HBA1:c.223G > C*), presenting abnormal Hb in zones F and **(C, B)** Sequencing diagram of *αα/−α^4^.^2^Hb Q-Thailand*. **(C)** Electrophoresis pattern of *−−SEA/−α^4^.^2^Hb Q-Thailand*, with abnormal Hb bands in zones F, Z15 (HbH), and Z12 (Hb Bart’s). **(D)** Sequencing diagram of *−−SEA/−α^4^.^2^ Hb Q-Thailand*. **(E)** Electrophoresis pattern of *Hb G-Honolulu* (*HBA2:c.91G > C*) with abnormal Hb in zone **(D) (F)** Sequencing diagram of *Hb G-Honolulu*. **(G)** Electrophoresis pattern of *Hb Hekinan* (*HBA1:c.84G > T*). **(H)** TGS sequencing diagram of *Hb Hekinan*. **(I)** Sequencing diagram of *Gγ-158C > T* and *Aγ-158C > T* variants.

Rare Hb structural variant genotypes within the *α-globin* gene cluster included two cases of −α*^4.2-Hb Q-Thailand^/αα* (**Figures 3A–B**), one case of −α*^4.2-Hb Q-Thailand^/−*α*^3.7^*, and one case of −α*^4.2-Hb Q-Thailand^/−−SEA* (**Figures 3C–D**). Additionally, one case of αα/ααHb G-Honolulu (*HbA2*: c.91 G > C) was identified (**Figures 3E–F**), along with one case of αα/ααHb Hekinan (*HbA1*: c.84 G > T) coexisting with Gγ-158C > T and Aγ-158C > T variants (**Figures 3G–I**).

#### 3.3.6 Structural hemoglobin variants in the *β-globin* cluster

**Figure 4:**
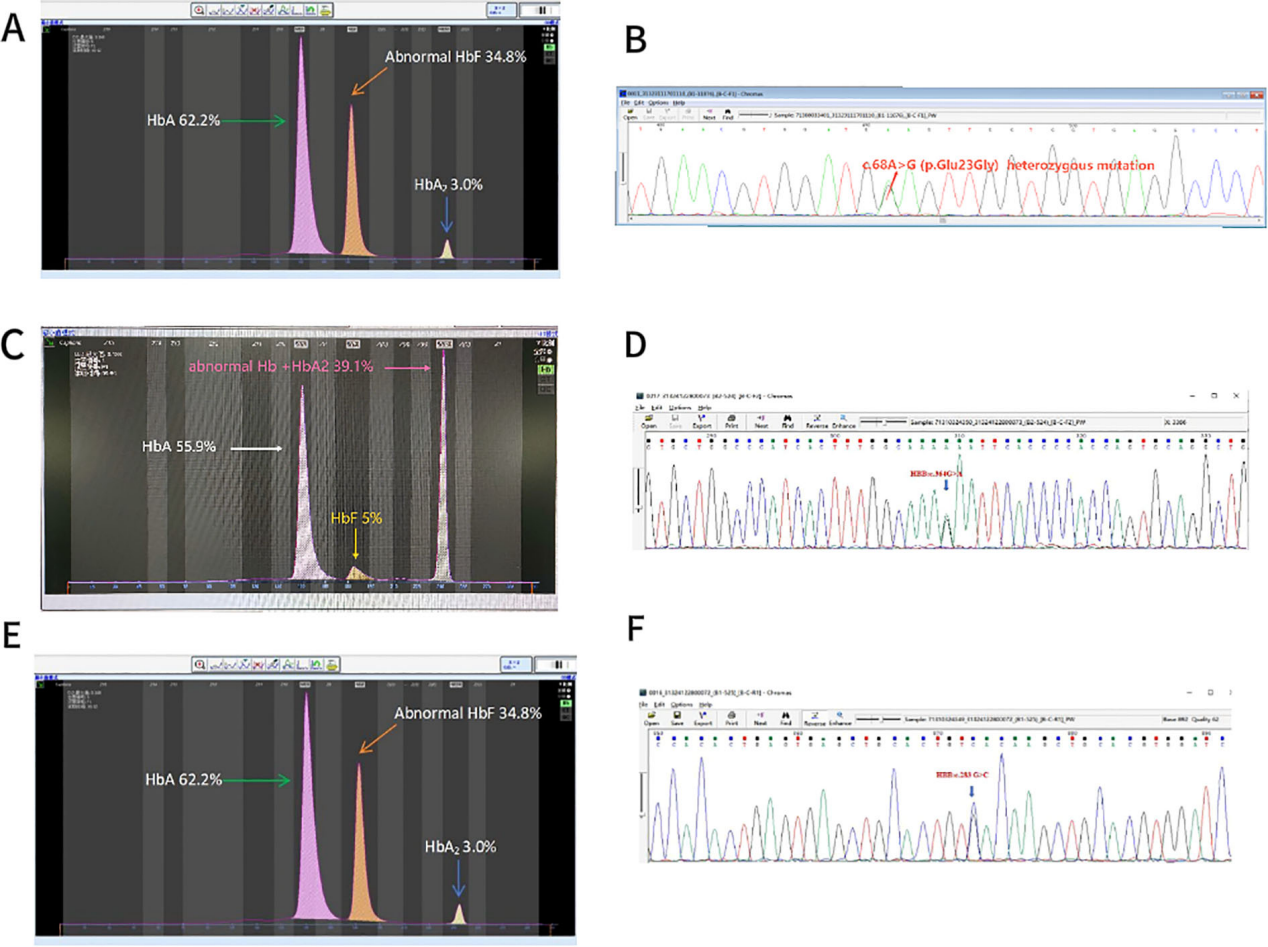
Rare hemoglobin structural variants in the *β-globin* gene cluster. **(A)** Electrophoresis pattern of *Hb G-Taipei* (*HBB:c.68A* > *G*), presenting abnormal Hb in zone D. **(B)** Sequencing diagram of *Hb G-Taipei*. **(C)** Electrophoresis pattern of *HbO-Arab* (*HBB:c.364G* > *A*). **(D)** Sequencing diagram of *HbO-Arab*. **(E)** Electrophoresis pattern of *Hb Barcelona* (*HBB:c.283G* > *C*). **(F)** Sequencing diagram of *Hb Barcelona*.

Within the *β-globin* gene cluster, Hb New York (*HBB*: c.341T > A) was the most frequently detected variant, identified in 13 cases. Additional variants included four cases of Hb J-Bangkok (*HBB*: c.170G > A), and single cases of Hb G-Taipei (HBB: c.68A > G) (**Figures 4A–B**), Hb O-Arab (*HBB*: c.364G > A) (**Figures 4C–D**), and Hb Barcelona (*HBB*: c.283G > C) (**Figures 4E–F**).

#### 3.3.7 δ-thalassemia variants

Two cases of δ-thalassemia CD10 (−G) (HBD: c.31delG) were identified. An additional case exhibited compound mutations involving δ-thalassemia CD65 (A > T) and CD115 (C > T), along with α-thalassemia ααCS/ααIVS-II-55 genotype.

### 3.4 Group B – genotype–phenotype discordance

Retrospective analysis of clinical records in Group B identified 13 cases exhibiting genotype–phenotype discordance. Subsequent rare thalassemia gene analysis confirmed the presence of variants in 9 of these cases.

## 4 Discussion

### 4.1 Rare variants and analysis of *α-globin* gene cluster CNVs

#### 4.1.1 2.4 deletion

A case with the -α2.4/αα genotype was identified. Previous reports by researchers in China characterized this deletion as involving a minimal genomic fragment, commonly associated with microcytic hypochromia and reduced HbA2 levels, but typically without apparent clinical manifestations (7, 8). However, when co-inherited with the αα/–SEA, the resulting -α2.4/–SEA combination may lead to a deletional form of HbH disease, which can manifest as mild to moderate anemia. Clinical management in such cases necessitates individualized genetic counseling and risk assessment during pregnancy.

#### 4.1.2 -α_90-93_(-8bp) (AGCTTCGG) mutation

Two cases of the rare SEA/−α_90-93_ (−8& bp)(AGCTTCGG) mutation were identified. The first reported instance of the αα/−α_90-93_(−8& bp)(AGCTTCGG) genotype in Guangxi was documented by Li et al. (9) DNA sequencing localized the 8& bp deletion to the *α2* gene, spanning nucleotides 34162 to 34171, and the mutation has been predicted to result in α^+^-thalassemia. When present in compound heterozygosity with the SEA deletion, this genotype leads to HbH disease, typically characterized by the presence of HbH and HbBart’s bands on hemoglobin electrophoresis. The clinical phenotype is similar to that observed in non-deletional HbH disease. Participants with the αα^CS^−−SEA genotype generally do not require regular transfusion support, though prenatal diagnostic evaluation is recommended. NGS is an appropriate method for detecting such mutations.

#### 4.1.3 HS-40 deletion

Two cases of the rare –HS-40/-α3.7 genotype were identified. Heterozygous deletion of the HS-40 regulatory region is typically associated with mild anemia. When co-inherited with the −α3.7 deletion, the resulting genotype may lead to HbH disease. Along with the SEA deletion, this genotype has been linked to Bart’s hydrops fetalis (10–12).

#### 4.1.4 First report of 4.3 deletion

A 44-year-old male, initially diagnosed with the conventional thalassemia genotype αα/–SEA, was re-evaluated using long-read sequencing conducted on the CycloneSEQ WT-02 single-molecule nanopore sequencer developed by BGI Genomics. The sequencing results identified a −-α4.3/–SEA genotype. This specific deletion has not been previously reported in the literature.

#### 4.1.5 α duplication

A 30-year-old female was identified as carrying an *α-globin* gene duplication, confirmed through testing at Berry TGS. The duplication spanned chr16:173490–175917, encompassing a 2.427& kb segment between the *HBA2* and *HBA1* genes. No previous reports have documented duplications within this specific region.

Rare CNVs within the *α-globin* gene cluster are typically associated with decreased HbA2 levels and are often accompanied by microcytic hypochromic features. The presence of HbH and/or HbBart’s bands on hemoglobin electrophoresis is indicative of HbH disease.

### 4.2 Rare variants and analysis of CNVs in the *β-globin* gene cluster

#### 4.2.1 Taiwan type

The three most common deletion-associated β-thalassemia variants in China, caused by large-scale deletions within the *β-globin* gene cluster, include Chinese type *^G^γ^+^(^A^γδβ)^0^* thalassemia, the SEA-Hereditary Persistence of Fetal Hemoglobin (HPFH), and the Taiwan type. These variants are typically characterized by microcytic hypochromic anemia accompanied by significantly elevated HbF levels. In this study, one adult male heterozygote with the *β*_Taiwanese_/*β*_N_ genotype (Taiwan type) was identified using GAP-PCR, consistent with previous findings reported by several members of the study team (13–15). Wang et al. documented five cases of the Taiwan type in Huadu, Guangdong Province, noting that mean HbA2 levels were higher than those observed in participants with Chinese type or SEA, whereas HbF levels were comparatively lower (15). Clinically, the presence of microcytic hypochromic anemia, elevated HbA2, and increased HbF levels along with negative results from conventional genetic testing should prompt the use of GAP-PCR to differentiate among the Chinese type, SEA, and Taiwan type.

#### 4.2.2 Southeast Asian deletion SEA-*HPFH*

An adult male in this study was diagnosed with β_17_/β_SEA-HPFH_ via Gap-PCR. Phenotypic descriptions of β-thalassemia associated with the SEA-*HPFH* deletion predominantly indicate mild to moderate anemia, with long-term survival generally not requiring regular blood transfusion support (16, 17).

Conventional genetic techniques are limited in detecting CNVs within the *β-globin* gene cluster. Accordingly, the integration of GAP-PCR with other diagnostic approaches and hematological parameters is recommended to achieve a comprehensive genetic and phenotypic assessment.

### 4.3 Rare variants and analysis of *α-globin* gene cluster SNVs

#### 4.3.1 ααCD30 heterozygote

The αα_CD30_ (*HBA2*: c.91_93delGAG) heterozygous variant is a rare mutation located within exon 1 of the HBA2 gene, affecting protein translation. The resulting clinical phenotype, *α*^+^→α^0^, has been documented by multiple members of the study team across southeastern, southwestern, and southern regions of China (5, 6, 8, 18). Lin et al. reported 12 cases exhibiting microcytic hypochromic anemia and reduced HbA2 levels (8). In this study, an adult male was identified with moderate anemia, significantly low HbA2, a prominent presence of HbH bands, and a small number of HbBart’s bands. The rare genotype αα_CD30_/–SEA was found, consistent with findings reported by Ren et al. and Feng et al., and was classified as HbH disease (19, 20).

#### 4.3.2 HBA2: c.168dup heterozygous variation

One case of the relatively rare HBA2 SNV, HBA2: c.168dup, was identified in this study. This variant, also referred to as codon 55/56 (+T), was previously reported in the heterozygous state by Peng et al. (6)

#### 4.3.3 Intron variation

A case of ααIVS-I-117(G > A)/αα was identified in this study, presenting without anemia or microcytic hypochromic features, consistent with a previously reported case by Lin et al. in Guangxi (8). The IVS-II-55(T > G) heterozygous variant has been documented in multiple studies, typically in the absence of anemia or microcytic hypochromia, and is often associated with slightly reduced *HbA2* levels (8, 21). This variant is frequently observed in combination with IVS-II-119(−G) and the SEA heterozygote in individuals lacking a clinical phenotype (22). In this study, one case of IVS-II-55(T > G) was detected in conjunction with a CS point mutation and δ-thalassemia. The IVS-II-34 (*HBA2*: c.300 + 34G > A) heterozygote is rarely reported in the literature; however, a case was identified in this study, with hematological parameters comparable to those described by Lin and Peng (8, 23).

#### 4.3.4 PolyA (AATAAA > AATAAG) (*HBA2*:c. *94A > G)

*PolyA* mutations arise from various base substitutions or deletions within the *HBA2* globin gene (24). Baysal et al. analyzed 84 chromosomes carrying *α*-thalassemia in the United Arab Emirates and reported that 47.4% harbored *PolyA1* mutations (25). This mutation is considered extremely rare in China. Recently, Zhuang et al. described an individual with *α^+^*-thalassemia carrying the *αPolyA1* mutation, who exhibited normal MCV (84& fL) and hemoglobin levels (113×10¹²g/L), with a mildly reduced MCH (26.5& pg) (26). In this study, one patient with this mutation along with the *βCD26 (GAG > AAG)* heterozygous variant, resulted in an imbalance of *α/β* globin chain synthesis. Notably, this patient did not exhibit anemia or microcytic hypochromic features. Hemoglobin electrophoresis data were not available for this case.

### 4.4 Rare variants and analysis of *β-globin* gene cluster SNVs

#### 4.4.1 *β*^N^/*β*^CD30^ (A > G) (*HBB c.91A > G*)

In this study, two adult women and one 7-year-old girl were identified as heterozygous for the *c.91A > G* mutation. All three participants presented with mild anemia, microcytic hypochromic features, and elevated HbA2 levels. Similar findings were reported by Luo (7). The *c.91A > G* mutation affects the sequence upstream of the 5′ splice junction of the first intron, disrupting normal mRNA splicing and resulting in a *β^0^* thalassemia.

#### 4.4.2 β^N^/β^IVS II-5^ (G > C) (HBB: c.315 + 5G > C)

The *IVS-II-5* (G > C) mutation, located at the fifth nucleotide of intron 2 in the *β-globin* gene, is classified as β^+^ thalassemia. In this study, two adult cases were identified with microcytic hypochromic features in the absence of anemia. Comparable cases have been documented by Yang and Ouyang (27, 28).

#### 4.4.3 β^N^/*β* IVS-II-81C

Multiple reports in the literature indicated that participants heterozygous for this variant typically do not exhibit anemia or microcytic hypochromia and often present with normal or elevated HbA_2_ levels (14, 28). In this study, one case was identified in which this variant coexisted with the SEA heterozygote, resulting in a mitigation of the α/β imbalance and the absence of a clinical phenotype.

#### 4.4.4 βN/βIVS-II-672 (*HBB* c.316-179)

No anemia, microcytic hypochromia, or abnormalities in HbA_2_ levels were observed. Similar findings have been documented by Huang et al. and Zhuang et al. (14, 26)

#### 4.4.5 β^N^/β^-31^ (A > C) (*HBB* c.-81A > C)

This heterozygous variant was identified in an adult female who exhibited no anemia or microcytic hypochromia and presented with an elevated HbA2 level, consistent with findings reported by Lin and Peng (18, 23). The c.-81A > C mutation is located within the 5′ untranslated region of the *HBB* gene, a non-coding region implicated in the regulation of mRNA stability, pre-mRNA splicing, and translation initiation. Disruptions in this region may contribute to the pathogenesis of thalassemia.

The microcytic hypochromic features and reduced HbA2 levels commonly observed in SNVs of the *α-globin* gene cluster are consistent with those seen in CNVs. Additionally, elevated HbA2 levels associated with SNVs in the *β-globin* gene cluster are frequently used as an initial screening indicator for α-thalassemia prior to confirmatory genetic testing.

### 4.5 Hong Kong type and triad

Hong Kong type and triad represent distinct forms of α-thalassemia. Hematological parameters in carriers of the HKαα/αα and anti-HKαα/αα genotypes typically fall within normal reference ranges. Laboratory identification is primarily based on GAP-PCR followed by agarose gel electrophoresis, where the presence of two bands (a normal 3.7& kb band and a weaker 3.7& kb band) suggests the Hong Kong type genotype (e.g., HKαα/αα, HKαα/−α3.7, or αα/−α3.7), while the presence of three bands (normal, 3.7, and SEA) indicates a likely combination of the Hong Kong type with the SEA heterozygote (29–31).

In complex cases involving the Hong Kong type, triad, 3.7& kb, and 4.2& kb deletions, confirmation through nested PCR combined with MLPA or TGS is essential. In this study, hematological parameters for individuals with the HKαα/αα genotype were within the normal range, consistent with findings reported by Huang et al. (14) One case of HKαα/ααCS presented with slightly reduced MCH and normal HbA_2_ levels. Similarly, Lin reported two cases with mildly reduced MCH and HbA2 levels as low as 1.5% (11). Hematological profiles of the HKαα/−−SEA genotype were nearly indistinguishable from those observed in αα/−−SEA carriers, aligning with the reports of Lin (8), Huang (14), and Liang (31).

From these findings, when one partner carries the αα/−−SEA genotype and the other carries HKαα/α, prenatal diagnostic testing is not considered necessary. The phenotype of the HKαα/−−SEA genotype differs significantly from those of αα3.7/−−SEA and αα4.2/−−SEA, both of which are associated with mild to moderate anemia. In cases where the fetus is diagnosed with either the αα3.7/−−SEA or αα4.2/−−SEA genotype, further counseling and discussion regarding pregnancy outcomes should be conducted with the pregnant individual and their family.

The *α-globin* gene cluster contains three homologous sequence elements like X-, Y-, and Z-box fragments. During meiosis, misalignment and unequal homologous recombination among these regions can result in triplication of the *α-globin* gene. Recombination between the homologous Z2 and Z1 boxes generates the −α3.7 single-gene deletion allele and the corresponding αααanti-3.7 triplication allele. Similarly, recombination between the X2 and X1 boxes produces the −α4.2 deletion allele and the αααanti-4.2 triplication allele. Conventional PCR-based detection techniques may misclassify αααanti-4.2 and αααanti-3.7 alleles as αα, as these alleles retain intact α2 and α1 genes. When co-inherited with β-thalassemia, the resulting imbalance in the β/α globin chain ratio can exacerbate the clinical severity, potentially leading to intermediate β-thalassemia (12).

In this group, no participants with αααanti-3.7/αα or αααanti-4.2/αα genotypes were identified. However, four cases were detected with complex triplication-associated β-thalassemia, including two individuals with β41–42/αααaaa3.7 and two with β17/αααaaa4.2 genotypes. All four cases presented with clinical TI and required blood transfusion. These findings are consistent with those reported by Ren et al. (32)

### 4.6 Rare Hb variants

#### 4.6.1 Rare α Hb variants

Hb Q-Thailand *(HBA1:* c.223G > C*)* arises from a GAC→CAC substitution at codon 74 of the *HBA1* gene, resulting in the replacement of aspartic acid with histidine at the N-terminal region of the α1-globin chain. This variant is frequently associated with the −α4.2 deletion type of thalassemia. Clinically, Hb Q-Thailand is often asymptomatic and may be misclassified as a simple −α4.2 deletion, potentially leading to missed diagnoses. Previous studies have identified Hb Q-Thailand as the most prevalent hemoglobin variant in Shaoguan, Guangdong (0.17%, 17/10,285), particularly among the Hakka population in Meizhou, Guangdong Province, and it is also widely distributed across Southeast Asia (33).

In this group, two cases of −α4.2-Hb Q-Thailand/αα were detected, without clinical anemia, microcytic hypochromia, or elevated HbA2 levels. Abnormal hemoglobin bands were observed in the F and C zones on capillary electrophoresis, consistent with prior reports (5, 8, 20, 27). However, in complex genotypes involving large-fragment deletions such as co-inheritance with the SEA deletion (−α4.2-Hb Q-Thailand/−−SEA) HbH disease may develop. In such cases, capillary electrophoresis indicates the absence of the HbA band, with abnormal bands appearing in the F, Z15 (HbH), and Z12 (Hb Bart’s) zones. According to the research, anemia associated with the HbH-4.2Q Thailand genotype may be more severe than that observed in HbH-WS, and comparable to deletional HbH disease (e.g., −α4.2/−−SEA). In this study, a pregnant participant with the −α4.2-Hb Q-Thailand/−−SEA genotype had a hemoglobin level of 79& g/L, excluding iron deficiency anemia associated with pregnancy (11).

Hb G-Honolulu (*HbA2*: c.91G > C) involves a glutamine-to-glutamic acid substitution at codon 31 of the α-globin chain, resulting in altered retention time (RT) of hemoglobin subcomponents during specific cation-exchange high-performance liquid chromatography (HPLC) assays. Previous studies have indicated that this variant may interfere with HbA1c measurement, occasionally producing an “E window” rather than a numerical value (34). In such cases, the E window is smaller than that typically observed with the Hb E variant, suggesting the presence of an alternative hemoglobin variant. Subsequent genetic testing confirmed the presence of Hb G-Honolulu. In this study, the abnormal hemoglobin band corresponding to this variant was located in the D zone. The absence of clinical anemia and microcytic hypochromia was consistent with findings reported by Luo and Yang (11, 27).

Hb Hekinan (*HbA1*: c.84G > T) was first described in Japan in 1988 and results from a point mutation at codon 27 of the *HBA1* gene, replacing glutamate with aspartate. Due to the absence of a net charge change, this variant is detectable by HPLC but may not be identified by capillary or cellulose acetate electrophoresis, increasing the risk of missed diagnosis. In this study, the variant was detected by TGS and was associated with elevated HbF levels resulting from γ-thalassemia, despite the absence of conventional thalassemia gene mutations. Chen reported a family with the Hb Hekinan variant in combination with the αα/−−SEA genotype, without significant hematological abnormalities or clinical anemia (35). It was hypothesized that the mutation in the *HBA1* gene characterized by reduced functional output triggered compensatory upregulation of the *HBA2* gene, partially offsetting α1 chain deficiency and resulting in a mild clinical phenotype. In this study, Hb Hekinan was associated with significantly elevated HbF levels due to coexisting Gγ-158C > T and Aγ-158C > T mutations, without anemia or microcytic hypochromia, though *HbA_2_* levels were reduced. No previous reports have documented this specific complex phenotype.

#### 4.6.2 Rare β-Hb variants

Hb New York (*HBB*: c.341T > A) is a hemoglobin variant characterized by the substitution of valine with glutamic acid at position 113 of the *β-globin* chain [β113 (G15) Val→Glu, GTG > GAG]. This replacement of a hydrophobic residue with an acidic one reduces the overall hydrophobicity of the β-chain and results in a structurally less stable hemoglobin molecule compared to Hb A, thereby increasing its susceptibility to degradation. Despite its relative frequency, Hb New York is typically not associated with significant hematological abnormalities or clinical anemia. On capillary hemoglobin electrophoresis, this variant presents as an abnormal band in the Z11 zone. Among the heterozygous individuals identified in this study, the mean concentration of abnormal hemoglobin was 43.05& ±& 3.96%, consistent with findings from multiple prior studies (20, 27, 28, 33, 36).

Hb J-Bangkok (*HBB*: c.170G > A) results from a point mutation at codon 56 of the *β-globin gene* (GGC > GAC), leading to the substitution of glycine with aspartic acid. This amino acid replacement induces a conformational change in the β-globin chain. Hb J-Bangkok is the second most common hemoglobin variant following HbE and Hb New York among heterozygotes. Clinically, its presentation is similar to that of Hb New York. On capillary electrophoresis, Hb J-Bangkok is observed as an abnormal hemoglobin band overlapping with the HbA_2_ band in the Z12 zone. In this study, four heterozygous cases of Hb J-Bangkok were identified, with an abnormal hemoglobin concentration of 43.05& ±& 3.96%, consistent with data reported in earlier studies (5, 11, 20, 27, 33).

Hb G-Taipei (*HBB*: c.68A > G) is a point mutation at codon 22 of the *β-globin* gene, resulting in the substitution of glutamic acid with glycine (Glu→Gly). HPLC analysis of HbA1c in participants carrying this variant has presented a RT longer than that of HbA_0_, attributed to the increased positive charge introduced by the mutation, which leads to an apparent elevation in HbA1c content (37). In this study, capillary electrophoresis of an Hb G-Taipei heterozygote demonstrated that 42.7% of the abnormal hemoglobin bands were located in the D zone. No clinical signs of anemia or hematological abnormalities were observed. These findings are consistent with reports from previous studies (27, 33).

HbO-Arab (*HBB*: c.364G > A) is a rare β-globin variant resulting from a substitution of glutamic acid with lysine at codon 121 (β121 Glu→Lys), effectively replacing normal *HbA*. Heterozygous carriers of HbO-Arab typically present with normal hemoglobin levels (38). In this study, one case of HbO-Arab was identified, with abnormal hemoglobin bands overlapping both HbA_2_ and HbF regions on capillary electrophoresis. Miniar Kalai et al. reported that individuals homozygous for HbO-Arab exhibited mild to moderate anemia (38). Van Gammeren et al. described a newborn carrying both HbO-Arab (*HBB*: c.364G > A) and Hb D-Los Angeles (*HBB*: c.664G > C), who developed mild microcytic anemia within one year (39). Elbashir et al. reported 13 African American individuals aged 2.7 to 62.5 years with Hb S/O, all of whom presented with hemolytic anemia (40). These findings indicate that the clinical phenotype associated with HbO-Arab is highly variable and may become more severe when co-inherited with other hemoglobin variants or β-thalassemia, often exceeding the clinical impact of the three more common Hb variants.

Hb Barcelona (*HBB*: c.283G > C) is a rare hemoglobin variant caused by the substitution of aspartic acid with histidine at position 94 of the β-globin chain. This mutation was first described by Aguilar et al. in a Spanish family presenting with mild polycythemia (41). Functional studies by Phillips et al. demonstrated that Hb Barcelona exhibits approximately a two-fold increase in oxygen affinity compared to normal HbA, likely accounting for the polycythemia observed in carriers without associated anemia (42). In this study, 34.8% of abnormal hemoglobin was detected in a case of Hb Barcelona, closely matching the 37% reported by Phillips et al. (42)

A common characteristic of abnormal hemoglobin variants, if involving *α-* or *β-globin* chains, is the appearance of abnormal bands on hemoglobin electrophoresis. These bands may vary in detectability; while some are readily identified, others are more subtle and may require confirmation through NGS or TGS. Unlike typical thalassemia syndromes, abnormal hemoglobinopathies do not consistently present with microcytic hypochromic features, which helps in their differential diagnosis. Furthermore, it should be noted that Hb Q-Thailand is frequently associated with the −α4.2 deletion. When co-inherited with the 4.2 deletion interlocking, the resulting genotype may resemble HbH disease, increasing the risk of missed diagnosis if not appropriately investigated.

### 4.7 δ-thalassemia

δ-thalassemia is primarily caused by point mutations. Both δ^0^- and δ^+^-thalassemia, if in the homozygous or heterozygous state, generally do not produce clinical symptoms. The principal hematologic feature is a reduced HbA_2_ level observed during hemoglobin analysis. To date, 19 distinct mutation types have been reported, including −77 T > C, −65 A > C, and CD30 G > C (43). Zhang et al. reported a δ-thalassemia prevalence of 0.49% in the Yunnan population, which is slightly higher than that observed in other southern Chinese populations (0.4%) (44).

In this study, *δ-globin* gene mutations were identified in 87 of 195 samples (44.62%). The most frequently observed mutation was −77 T > C, accounting for 88.51% of cases (77/87), followed by −30 T > C (3.45%, 3/87) and a start codon mutation (Met > Ile) (2.30%, 2/87). Two heterozygous cases were detected with the *HBD*:c.31delG mutation, both presenting with an HbA2 level of 1.4%. Additionally, one case of α-thalassemia with the genotype ααCS/ααIVS-II-55 was found to co-occur with δ-thalassemia mutations (CD65 A > T and CD115 C > T), exhibiting an even lower HbA2 level of 0.7% and no evidence of clinical anemia.

## 5 Conclusion

A total of 42 rare thalassemia gene mutations were identified in the Northern Guangxi population, including the first reported case of −α^4.3^/−−SEA, a rare 4.3& kb deletion (chr16:176935–181274DEL) encompassing the α_1_ gene and involving regional recombination with a non-functional −X–Y–Z segment. This finding contributes to the expanding mutational spectrum of thalassemia in the region. Each rare thalassemia variant was associated with distinct hematological characteristics and hemoglobin electrophoresis profiles. Clinically, rare genotypes may be inferred through integration of phenotypic features and corresponding hematological parameter patterns. The selection of appropriate diagnostic methods or a combination of multiple methods can improve diagnostic accuracy, reduce the likelihood of missed or incorrect diagnoses, and support effective prenatal and postnatal management strategies.

## Data Availability

The original contributions presented in the study are included in the article/**Supplementary Material**. Further inquiries can be directed to the corresponding author.
