# Correction: Genotypic and phenotypic characterization of rare globin variants in Northern Guangxi, China

**DOI:** 10.3389/fimmu.2026.1807339

**Published:** 2026-03-17

**Authors:** Wei-Jia Yang, Qing-Ping Kang, Li-Ming Liang, Qian Zhou, Xiao-Min Gong, Min Dou, Cui-Juan Huang, Ying Lin

**Affiliations:** 1 Department of Eugenics and Genetics, Guilin People’s Hospital, Guilin, Guangxi, China; 2 Genetic Metabolism Laboratory, Guilin Women and Children’s Hospital, Guilin, Guangxi, China; 3 Genetic and Precision Medicine Lab, The First Affiliated Hospital of Guilin Medical University, Guilin, Guangxi, China

**Keywords:** hemoglobin electrophoresis, phenotype, rare globin variation, genotype thalassemia, molecular diagnosis

Error in figure/table

The values of “HbA2 for HbOArab, HBB: c.364G>A; CD121 (GAA>AAA)” in Supplementary Material Table S5 were filled in incorrectly. The original table showed “0”, and they should be corrected to “ND (Not Detected)”. The corrected table is as follows.

In **Figure 4**, there is an error in 4C. The text “HbF or abnormal Hb 39.1%” on the original image should be changed to “abnormal Hb + HbA2 39.1%”. The text “HbA2 or abnormal 5%” on the original image should be changed to “HbF 5%”.

**Figure 4:**
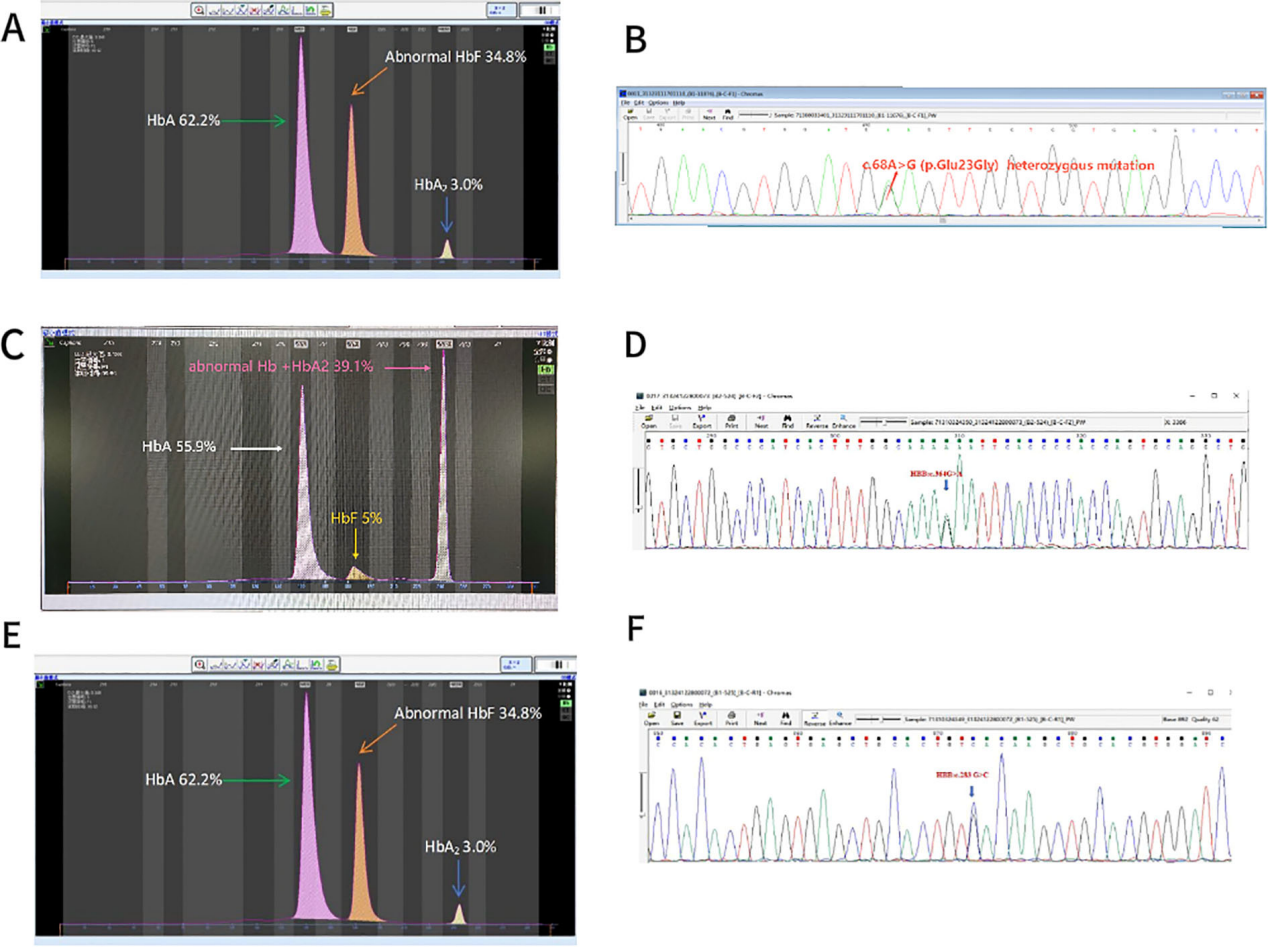
Rare hemoglobin structural variants in the β-globin gene cluster. **(A)** Electrophoresis pattern of *Hb G-Taipei* (*HBB:c.68A* > *G*), presenting abnormal Hb in zone D. **(B)** Sequencing diagram of *Hb G-Taipei*. **(C)** Electrophoresis pattern of *HbO-Arab* (*HBB:c.364G* > *A*). **(D)** Sequencing diagram of *HbO-Arab*. **(E)** Electrophoresis pattern of *Hb Barcelona* (*HBB:c.283G* > *C*). **(F)** Sequencing diagram of *Hb Barcelona*.

The original article has been updated.

