# Supplementary material for: Genotypic and phenotypic characterization of rare globin variants in Northern Guangxi, China
Source: Front Immunol. 2025 Oct 28;16:1695120. doi: 10.3389/fimmu.2025.1695120 (PMC12602246; doi:10.3389/fimmu.2025.1695120)
Supplement: Supplementary file 1 [file Table1.docx]

Table S1. Genotypes, hematological parameters and phenotypes of rare CNVs in the *α-* and *β-globin* gene clusters.

| Genotypes | No. | n | Sex | Age (year) | Hb  (g/L) | MCV  (fl) | MCH (g/L) | HbA (%) | HbA_2_ (%) | HbF (%) | Abnormal Hb (%) | Parameter characteristics | Phenotype |
| --- | --- | --- | --- | --- | --- | --- | --- | --- | --- | --- | --- | --- | --- |
| -α2.4/αα | 1 | 1 | Female | 30 | 127 | 79.9 | 26.6 | 98 | 2 | 0 | 0 | microcytic hypochromia,  reduced HbA2 | α+  thalassemia |
| HBA2:chr16:173490-175917 repeat 2.427Kb | 2 | 1 | Female | 30 | 132 | 85.9 | 27.9 | 97.6 | 2.4 | 0 | 0 | Reduced HbA2 | α+  thalassemia |
| βN/βTaiwanese | 3 | 1 | Male | 37 | 131 | 66.5 | 19.5 | 88.4 | 7.7 | 3.9 | 0 | microcytic hypochromia,  elevated HbA2 and HbF | β+  thalassemia |

Table S2. Genotypes, hematological parameters and phenotypes of rare SNVs in the *α-* and *β-globin* gene clusters.

| Genotypes | No. | n | Sex | Age (year) | Hb  (g/L) | MCV  (fl) | MCH (g/L) | HbA (%) | HbA_2_ (%) | HbF (%) | Abnormal Hb (%) | Parameter characteristics | Phenotype |
| --- | --- | --- | --- | --- | --- | --- | --- | --- | --- | --- | --- | --- | --- |
| --SEA/ααCD30  HBA2:c.91_93delGAG | 4 | 1 | Male | 33 | 95 | 61 | 19 | 66.5 | 0.5 | 0 | HbH:32.1, HbBart's 0.9 | HbH:32.1, HbBart's 0.9 | TI(HbH disease) |
| αα/ααHBA2:c.168dup | 5 | 1 | Male | 25 | 160 | 78.8 | 25.7 | 97.4 | 2.6 | 0 | 0 | Microcytic hypochromia | α+thalassemia |
| αα/ααIVS-I-117,  HBA1:c.96-1 G>A | 6 | 1 | Male | 30 | 164 | 81 | 25.9 | 97.6 | 2.4 | 0 | 0 | Microcytic hypochromia,  reduced HbA2 | α+thalassemia |
| αα/ααIVS-II-55  HBA2:c.300+55T>G | 7 | 1 | Female | 26 | 120 | 80 | 26 | 97.7 | 2.3 | 0 | 0 | Microcytic hypochromia,  reduced HbA2 | α+thalassemia |
| ααCS/ααIVS-II-55, with δthalassemiaCD65(A>T),CD115(C>T) | 8 | 1 | Female | 37 | 112 | 78.8 | 26.4 | 99.3 | 0.7 | 0 | 0 | Microcytic hypochromia,  lower HbA2 | α with δthalassemia |
| αα/ααIVS-II-34,  HBA2:c.300+34G>A | 9 | 1 | Female | 22 | 123 | 76.6 | 25.5 | 97.5 | 2.5 | 0 | 0 | Microcytic hypochromia, low normal value of HbA2 | α+thalassemia |
| αα/ααPoly A (AATAAA>AATAAG);HBA2:c.*94A>G,  βN/  βCD26(GAG>AAG) | 10 | 1 | Male | 57 | 142 | 76.5 | 26.1 | Data loss | Data loss | Data loss | Data loss | Abnormal band of glycosylated Hb | α with β |
| βN/βCD 30 (A>G),HBB:c.91A>G | 11 | 1 | Female | 31 | 101 | 9.3 | 20.8 | 94 | 5.3 | 0.7 | 0 | Microcytic hypochromia, elevated HbA2 | β0thalassemia |
| βN/βCD30 (A>G),HBB:c.91A>G | 12 | 1 | Female | 38 | 110 | 62.4 | 19.2 | 94 | 6.0 | 0 | 0 | Microcytic hypochromia, elevated HbA2 | β0thalassemia |
| βN/βCD 30 (A>G),HBB:c.91A>G | 13 | 1 | Female | 7 | 101 | 60 | 8.8 | 94.1 | 5.9 | 0 | 0 | Microcytic hypochromia, elevated HbA2 | β0thalassemia |
| βN/βIVS II-5(G>C) | 14 | 1 | Female | 36 | 125 | 80.5 | 25.6 | 96.2 | 3.8 | 0 | 0 | Microcytic hypochromia, elevated HbA2 | β+thalassemia |
| βN/βIVS II-5(G>C) | 15 | 1 | Male | 30 | 145 | 78.9 | 26.3 | 96 | 4 | 0 | 0 | Microcytic hypochromia, elevated HbA2 | β+thalassemia |
| βN/βIVS-Ⅱ-81C, αα/--SEA | 16 | 1 | Female | 25 | 125 | 64.4 | 19.7 | 96.95 | 3.05 | 0 | 0 | Microcytic hypochromia | α with β |
| βN/βIVS-Ⅱ-672(A>C),  HBB: C.316-179A>C | 17 | 1 | Female | 28 | 129 | 86.2 | 29.2 | 97.3 | 2.7 | 0 | 0 | Normal blood routine | β+thalassemia |
| βN/β-31(A>C),  HBB: C.-81A>C | 18 | 1 | Female | 26 | 120 | 84.9 | 27.5 | 94.2 | 4.1 | 1.7 | 0 | Normal blood routine, elevated HbA2 | β+thalassemia |

Table S3. Genotypes, hematological parameters and phenotypes of Hong Kong type and α triad.

| Genotypes | No. | n | Sex | Age (year) | Hb  (g/L) | MCV  (fl) | MCH (g/L) | HbA_2_ (%) | Parameter characteristics | Phenotype |
| --- | --- | --- | --- | --- | --- | --- | --- | --- | --- | --- |
| HKαα/αα | 19-20 | 3 | 1Female  2Male | 30.67±2.31 | 146.33±22.30 | 89.1±2.13 | 29.87±0.25 | 2.9±0.1 | Normal blood parameters | α+  thalassemia |
| HKαα/ααWS | 21-23 | 3 | Male | 29.33±5.51 | 162.67±6.51 | 86.57±6.33 | 28.27±1.90 | 2.77±0.25 | Normal blood parameters | α+  thalassemia |
| HKαα/ααCS | 24 | 1 | Female | 28 | 114 | 83.8 | 26.5 | 2.6 | Lower MCH | α+  thalassemia |
| HKαα/--SEA | 25-27 | 3 | Female | 34±5.66 | 118.5±3.54 | 69.4±0.28 | 16.3±7.64 | 2.35±0.07 | Microcytic hypochromia, reduced HbA2 | α0  thalassemia |
| β41-42/αααaaa3.7 | 28-29 | 2 | Female | 15.5±20.51 | 82.5±7.78 | 68.2±2.55 | 21.15±2.76 | 4.5±1.41 | Microcytic hypochromia, elevated HbA2 | TI |
| β17/αααaaa4.2 | 30 | 2 | 1Male  1Female | 30.5±6.36 | 80.5±3.54 | 76.75±6.01 | 23.45±0.64 | 4.35±0.50 | Microcytic hypochromia, elevated HbA2 | TI |

Table S4. Genotype, electrophoretic characteristics and phenotype of rare Hb structural variation in the *α-globin* gene clusters.

| Genotypes | No. | n | Sex | Age (year) | Hb  (g/L) | MCV  (fl) | MCH (g/L) | HbA(%) | HbA_2_(%) | HbF% | Abnormal Hb (%) | Parameter characteristics | Phenotype |
| --- | --- | --- | --- | --- | --- | --- | --- | --- | --- | --- | --- | --- | --- |
| -α4.2/αα,  Hb Q-Thailand | 31 | 1 | Female | 37 | 111 | 77.6 | 26.1 | 69.1 | 1.9 | 28.3 | Hb C zone 0.7 | Abnormal Hb located in F and C zones, microcytic hypochromia | α0  thalassemia |
| -α4.2/αα,  Hb Q-Thailand | 32 | 1 | Female | 28 | 114 | 78.5 | 25.2 | 67.7 | 1.8 | 29.8 | Hb C zone 0.7 | Abnormal Hb located in F and C zones, microcytic hypochromia | α0  thalassemia |
| -α4.2/-α3.7,  Hb Q-Thailand | 33 | 1 | Male | 30 | 137 | 72.1 | 21.6 | 56.4 | 1.1 | 41.7 | Hb Z1 0.8 | Abnormal Hb located in F and Z1 zones, microcytic hypochromia | α0  thalassemia |
| -α4.2/--SEA,  HbQ-Thailand | 34 | 1 | Female | 27 | 79 | 54.5 | 16.4 | 0 | 1.5 | 92.1 | HbH 5.7  Hb Bart's 0.7 | Abnormal Hb located in F, Z15 and Z12 zones, microcytic hypochromia | TI |
| αα/ααHb G-Honolulu;  HBA2: c.91 G>C | 35 | 1 | Male | 36 | 159 | 91 | 31 | 74.4 | 1.2 | 0.4 | 24 | Normal blood routine, abnormal Hb located in D zone | α+  thalassemia |
| Hb HekinanⅡ(HbA1:c.84G>C)Gγ-158 C>T,  Aγ-158 C>T | 36 | 1 | Female | 32 | 124 | 84.5 | 30 | 86.5 | 2.2 | 11.3 | 0 | Reduced HbA2, significantly elevated HbF; | α with γ |

Table S5. Genotypes, hematological parameters and phenotypes of rare Hb structural variation in the *β-globin* gene clusters.

| Genotypes | No. | n | Sex | Age (year) | Hb  (g/L) | MCV  (fl) | MCH (g/L) | HbA (%) | HbA_2_ (%) | HbF (%) | Abnormal Hb (%) | Parameter characteristics | Phenotype |
| --- | --- | --- | --- | --- | --- | --- | --- | --- | --- | --- | --- | --- | --- |
| Hb New York,  HBB:c.341T>A,  CD113( GTG->GAG) | 37-49 | 13 | 10Male  3Female | 35.31±6.05 | 142.69±15.84 | 89.46±6.03 | 29.94±2.12 | 53.9±4.29 | 2.75±0.78 | 0.29±0.63 | 43.05±3.96 | Abnormal Hb band | β+  thalassemia |
| Hb J-Bangkok  HBB:c.170G>A;  CD56(GGC->GAC) | 50-53 | 4 | Female | 30±5.48 | 117.25±10.37 | 85.73±14.21 | 27.48±5.84 | 46.6±0.83 | 2.38±0.28 | 0.05±0.10 | 50.98±0.76 | Abnormal Hb band | β+  thalassemia |
| HbGTaipei,HBB:c.68 A>G;CD22(GAA->GGA ) | 54 | 1 | Male | 45 | 147 | 79 | 31 | 54.6 | 2.7 | 0 | 42.7 | Abnormal Hb located in D zone | β+  thalassemia |
| HbOArab,HBB:c.364G>A;CD 121( GAA>AAA) | 55 | 1 | Female | 28 | 122 | 84 | 29.9 | 55.9 | 0 | 5 | 39.1 | Abnormal Hb band | β+  thalassemia |
| HbBarcelona,HBB:c.283G>C (p.Asp95His) | 56 | 1 | Female | 28 | 129 | 89.3 | 30.0 | 62.2 | 3.0 | 0 | 34.8 | Abnormal Hb band | β+  thalassemia |

Table S6. Genotypes, hematological parameters and phenotypes of rare SNVs in the *δ-globin* gene clusters.

| Genotypes | No. | n | Sex | Age (year) | Hb  (g/L) | MCV  (fl) | MCH (g/L) | HbA (%) | HbA_2_ (%) | HbF (%) | Abnormal Hb (%) | Parameter characteristics | Phenotype |
| --- | --- | --- | --- | --- | --- | --- | --- | --- | --- | --- | --- | --- | --- |
| Codon10(-G)in delta heterozygous variant, HBD:c.31delG | 57 | 1 | Male | 34 | 164 | 93.9 | 31.4 | 98.6 | 1.4 | 0 | 0 | Reduced HbA2 | δthalassemia heterozygote |
| Codon10(-G)in delta heterozygous variant, HBD:c.31delG | 58 | 1 | Male | 29 | 180 | 83.1 | 27.4 | 98.6 | 1.4 | 0 | 0 | Reduced HbA2 | δthalassemia heterozygote |
| αα/ααIVS-II-55, with δthalassemiaCD65(A>T),CD115(C>T) | 8 | 1 | Female | 37 | 112 | 28.8 | 26.4 | 99.3 | 0.7 | 0 | 0 | Significantly reduced HbA2 | α with δthalassemia |

Table S7. Genotypes and hematological parameters of rare thalassemia in 9 cases with genotype–phenotype discordance.

| Genotypes | No. | n | Sex | Age (year) | Hb  (g/L) | MCV  (fl) | MCH (g/L) | HbA (%) | HbA_2_ (%) | HbF (%) | Abnormal Hb (%) | Parameter characteristics | Phenotype |
| --- | --- | --- | --- | --- | --- | --- | --- | --- | --- | --- | --- | --- | --- |
| --SEA/-αCD90-93(-8bp)(-AGCTTCGG) mutation | 59 | 1 | Male | 6 | 74 | 80.2 | 22.2 | 73.3 | 0.5 | 0 | 0 | HbH 25.4,  HbBart's 0.8 | TI  (HbH disease) |
| --SEA/-αCD90-93(-8bp)(-AGCTTCGG) mutation | 60 | 1 | Male | 3 | 89 | 74.6 | 21.1 | 72.3 | 0.5 | 0 | 0 | HbH 25.8  HbBart's 1.4 | TI  (HbH disease) |
| aa/-αCD90-93(-8bp)(-AGCTTCGG) mutation | 61 | 1 | Male | 41 | 136 | 82.1 | 26 | 97.6 | 2.4 | 0 | 0 | MCH、HbA_2_ on the edge of normal low value | α0thalassemia |
| -3.7/--HS-40 | 62 | 1 | Male | 11 | 81 | 58.8 | 17.2 | 92.2 | 2.0 | 0.5 | HbH 5.3 | HbH 5.3 | TI  (HbH disease) |
| αα/--HS-40 | 63 | 1 | Male | 40 | 121 | 68.7 | 19.6 | 97.7 | 2.3 | 0 | 0 | microcytic hypochromia, low HbA2 | α0thalassemia |
| -α4.3/--SEA | 64 | 1 | Male | 44 | 44-85 | 58 | 18 | 81.1 | 0.6 | 0 | 0 | HbH 19.1,  HbBart's 0.6 | TI  (HbH disease) |
| βIVS-Ⅱ-654/βc.91A>G(trans-position) | 65 | 1 | Female | 3 | 85 | 81.3 | 24.8 | 91.7 | 2.6 | 5.7 | 0 | High HbF | TM |
| β17/βSEA-HPFH | 66 | 1 | Male | 43 | 93 | 71.6 | 23.7 | 0 | 3.7 | 96.3 | None | Significant elevated HbF | TI |
| βCD17/βIVS II-5(G>C) | 67 | 1 | Female | 41 | 58 | 61 | 19 | 75.5 | 5.8 | 18.7 | None | Significant elevated HbF | TI |
